# Efficacy, safety and cost analyses in ulcerative colitis patients undergoing granulocyte and monocyte adsorption or receiving prednisolone

**DOI:** 10.1186/1471-230X-13-41

**Published:** 2013-03-01

**Authors:** Keiichi Tominaga, Masakazu Nakano, Mina Hoshino, Kazunari Kanke, Hideyuki Hiraishi

**Affiliations:** 1 Department of Gastroenterology, Dokkyo Medical University, 880, Kitakobayashi, Mibu, Shimotsuga, Tochigi, 321-0293, Japan

**Keywords:** Ulcerative colitis, Adsorptive granulocyte and monocyte apheresis, Prednisolone, Medical cost, Treatment safety

## Abstract

**Background:**

Patients with ulcerative colitis (UC) are treated with prednisolone (PSL), which causes adverse side effects. Extracorporeal granulocyte/monocyte adsorption (GMA) with an Adacolumn depletes elevated/activated myeloid lineage leucocytes as sources of inflammatory cytokines. We were interested to evaluate the efficacy, safety and the treatment cost for PSL and GMA.

**Methods:**

Forty-one patients with active UC had achieved remission with GMA, at 1 or 2 sessions/week, up to 10 sessions (n=24) or with orally administered PSL (1mg/kg bodyweight, n=17). Clinical activity index (CAI) ≤4 was considered clinical remission. Following remission, patients received 5-aminosalicylic acid (2250-3000mg/day) or sulphasalazine (4000-6000mg/day) as maintenance therapy and were followed for 600 days. The total treatment cost was assessed based on 1€=150JPY.

**Results:**

PSL was tapered after two weeks, and discontinued when a patient achieved remission. The average time to the disappearance of at least one major UC symptom (haematochezia, diarrhoea, or abdominal discomfort) was 15.3 days in the GMA group and 12.7 days in the PSL group, while time to remission was 27.9 days in the GMA group and 27.6 days in the PSL group, CAI 0.8 and 2.0, respectively. The Kaplan-Meier plots showed similar remission maintenance rates over the 600 days follow-up period. The average medical cost was 12739.4€/patient in the GMA group and 8751.3€ in the PSL group (P<0.05). In the GMA group, 5 transient adverse events were observed vs 10 steroid related adverse events in the PSL group (P<0.001).

**Conclusions:**

In appropriately selected patients, GMA has significant efficacy with no safety concern. The higher cost of GMA vs PSL should be compromised by good safety profile of this non-pharmacological treatment intervention.

## Background

Ulcerative colitis (UC) is one of the two major phenotypes of the chronic inflammatory bowel disease (IBD); the other major phenotype of IBD is Crohn’s disease [1,2]. UC afflicts millions of individuals throughout the world, causing symptoms like fever, weight loss, diarrhoea, rectal bleeding and abdominal discomfort, which impair individual’s activities and quality of life. Currently, there is no permanent cure for UC, the treatments focus on controlling the disease activity with pharmacologics, often at high doses over long periods of time [1,3,4]. Corticosteroids like prednisolone (PSL) have had a major role in the treatment of UC since the pioneering trials by Truelove, and colleagues several decades ago [5,6]. However, when used in chronic, remitting-relapsing conditions like UC, corticosteroids cause adverse side effects, steroid dependency and refractoriness [1,3]. It is known that long-term administration of corticosteroids potentially increases the development of osteoporosis, and cataracts as adverse side effects, which are more likely at higher corticosteroid doses [7-9]. Accordingly, in one major study, the incidence of severe adverse events was higher in the high dose PSL group as compared with the low dose group [9].

There is evolving evidence for myeloid lineage leucocytes (granulocytes and monocytes) as major factors in the immunopathogenesis of UC. Thus, patients with UC may harbour elevated peripheral neutrophils [10] in the presence of compromised lymphocytes [11-13]. Further, in UC, neutrophils show activation behavior [14] and prolonged survival [15]. Factors known to promote neutrophil survival include inflammatory cytokines [16], and paradoxically corticosteroids [17]. Indeed, neutrophil activation and prolonged survival is a feature of persistent intestinal inflammation and histological examinations of the mucosal tissue in biopsy specimens from patients with active UC reveals a spectrum of pathologic manifestations among which presence of neutrophils accounts not only for the morphologic lesions of UC, but also for the prevailing patterns of mucosal inflammation [1,14,15,18]. When activated, granulocytes together with monocytes/macrophages produce an array of pleiotropic cytokines like tumour necrosis factor (TNF)-α, interleukin (IL)-1β, IL-6, IL-12, IL-23 which are strongly inflammatory [19-23]. These have given rise to the inference that elevated and activated granulocytes and monocytes should be targets of therapy in UC. Based on theses observations, the Adacolumn has been developed for selective depletion of granulocytes and monocytes by adsorption (GMA).

In the early 2000, following a major multicentre clinical trial [24,25], GMA was introduced into the Japan national health reimbursement scheme as remission induction therapy for patients with UC. Subsequently, investigators reported high remission rates and good safety profile for GMA [26-30], but disappointing trial outcomes have been reported as well [31]. In this study, we aimed to assess the efficacy, safety and treatment cost in two cohorts of patients with active UC treated with GMA or with PSL as remission induction therapy.

## Methods

### Patients

The subjects were 41 patients with UC in the active stage who had been successfully treated by GMA as remission induction therapy in the period from April 2000 to March 2009 at our hospital (GMA group, n=24) or with the corticosteroid, prednisolone (PSL group, n=17). The demographic features of the included patients are presented in Table 1. These 41 patients were retrospectively reviewed. The patients were from two bigger cohorts, 43 and 24, respectively who had responded to these interventions. The remaining patients (19 and 7) either had not achieved remission or were not eligible for this follow-up study (receiving additional medications was one exclusion criterion). Following remission induction, patients were given 5-aminosalicylic acid (5-ASA, 2250-3000mg/day) or sulphasalazine (4000-6000mg/day) as maintenance therapy. If a patient had been on azathioprine (AZA) for >8 weeks prior to the start of the present therapy (GMA or PSL), the patient could be included and AZA was to be continued at the same dosage if required (Table 1). PSL dose was to be tapered within two weeks following the start of administration and subsequently discontinued when patient achieved remission.

**Table 1:** Entry demography of the 41 patients of this study

		**GMA**	**PSL**
**Gender:**	**Male/Female**	**14/10**	**7/10**
**Mean age (range)**		**33.6(15–78)**	**35.4(13–65)**
**Extent of UC**	**Total**	**17**	**10**
	**Left sided**	**6**	**5**
	**Recto-sigmoid**	**1**	**2**
**Clinical course**	**Chronic continuous type**	**0**	**1**
	**Relapsing remitting type**	**28**	**14**
	**First UC episode**	**0**	**2**
**Inpatient/Outpatient**		**19/5**	**17/0**
**Patients with a history of exposure to PSL**		**17**	**13**
**Patients on AZA (50mg/day)**		**8**	**8**

*GMA*, adsorptive granulocyte and monocyte apheresis; *PSL*, predonisolone; *AZA*, azathioprine (started ≥8 weeks ahead of entry).

All patients were included following a relapse of ulcerative colitis (UC)

### Adsorptive depletion of myeloid lineage leucocytes

Adsorptive depletion of myeloid lineage leucocytes (GMA) was done with the Adacolumn as previously described [10,12,13,28]. The Adacolumn is filled with cellulose acetate beads of 2mm in diameter as the column adsorptive leucocytapheresis carriers [32,33]. The carriers adsorb from the blood in the column most of the granulocytes and monocytes together with a significant fraction of platelets (FcγR and complement receptor bearing cells); lymphocytes are spared. In fact lymphocytes increase after GMA [12,13,32,33], including the CD4+CD25high+ phenotype, which is known as a key regulator of immune function [33,34]. Each patient received 1 or 2 GMA sessions/week, up to 10 sessions. One session was 60–90 min at 30mL/min [28].

### Treatment of patients in the PSL group

In the PSL group, patients received PSL orally, at 1mg/kg (bodyweight)/day orally as the starting dose. The PSL dose was to be tapered at 5 to 10 mg per week within 2 weeks following initiation of therapy. Following remission, PSL could be discontinued and replaced with maintenance therapy, 5-ASA or sulphasalazine (indicated above).

### Assessment of disease activity and treatment efficacy

UC clinical activity index (CAI) was determined at entry and at appropriate time points during active treatment and the 24 months follow-up period according to Rachmilewitz [35]. Factors considered in the present study for assessing efficacy were blood in the stool, diarrhoea and abdominal pain as major complication of UC that most seriously impact activities and quality of life [1,2]. The time to the cessation of at least one major UC symptom as well as the time to remission (CAI ≤4) was used to assess the immediate efficacy of the treatments, while the long-term effect on UC activity was assessed by the Kaplan-Meier survival graphs. The cumulative dose of PSL, average treatment cost per patient were also determined.

### Ethical considerations

In Japan, GMA with the Adacolumn is an officially approved therapeutic option for patients with active IBD. Additionally, informed consent was obtained from all patients after explaining the study aim and the nature of the procedures involved. Adherence was made to the Principle of Good Clinical Practice and the Declaration of Helsinki at all times.

### Statistical analysis

Where appropriate, data are presented as the mean ± SD values. The differences between sets of parametric data were analyzed by using the t-statistic. The relapse rate was estimated by the Kaplan-Meier survival graphs and the log-rank test. P<0.05 was considered significant.

## Results

### Time to the disappearance of at least one major UC symptom and clinical remission

**Figure 1:**
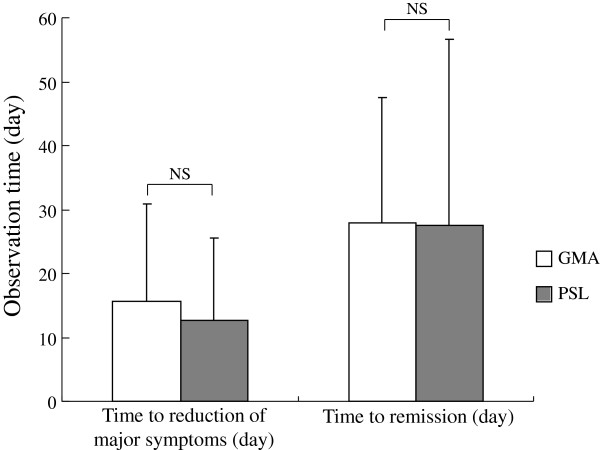
**This figure shows time to the disappearance of at least one major ulcerative colitis (UC) symptoms (diarrhoea, rectal bleeding, and abdominal discomfort) as well as time to remission (clinical activity index, CAI≤4) following the start of interventions.** As shown, no statistically significant (ns) difference between the patients who received the corticosteroid, PSL and those who received the non-pharmacologic, GMA was found.

Figure 1 shows time to the disappearance of at least one major UC complication like diarrhoea, haematochezia or abdominal discomfort as well as time to remission (CAI ≤4) in the two groups following start of interventions (GMA and PSL). As shown, no statistically significant difference between the patients who received PSL and those who received the non-pharmacologic, GMA was found. The average time to the cessation of at least one major UC complication was 15.6 ± 15.2 days in the GMA group and 12.7 ± 12.9 days in the PSL group. Likewise, the average time to clinical remission was 27.9 ± 19.6 days in the GMA group and 27.6 ± 29.0 days in the PSL group.

### Efficacy outcomes based on CAI

**Figure 2:**
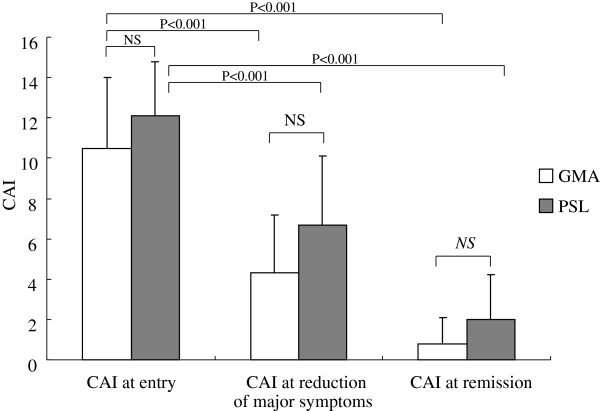
**Overall improvements in the clinical activity indices (CAI) in two groups of patients with active UC receiving GMA or PSL.** This figure shows average CAI values at baseline, at the time point when major UC symptoms were reduced and when patients achieved remission. No significant (ns) difference between the patients who received PSL and those who received the non-pharmacologic intervention, GMA was found.

The outcomes of the efficacy assessment based on CAI is presented in Figure 2, essentially echoing the outcomes seen in Figure 1. The average CAI value when at least one of the major complications of UC disappeared was 4.3 ± 2.9 in the GMA group and 6.7 ± 3.4 in the PSL group indicating that at this time point, clinical remission was not achieved by all patients. Instead, the CAI value was significantly reduced in both groups relative to active disease, but without any significant difference between the two groups. Similarly, the average CAI value when the patients achieved remission was 0.8 ± 1.3 in the GMA group and 2.0 ± 2.2 in the PSL group, without significant difference between the GAM and the PSL groups.

### The Kaplan-Meier graphs of remission maintenance

**Figure 3:**
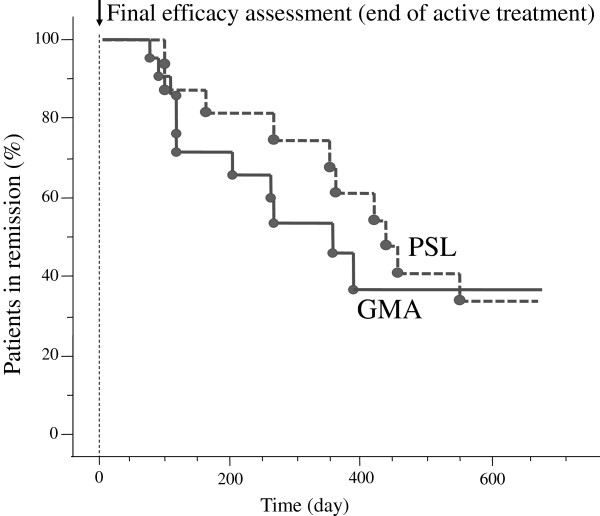
**The Kaplan-Meier plots showing maintenance of remission in two groups of patients receiving GMA or PSL.** Patients who achieved remission (CAI≤4) at the end of the active treatment period (arrow head) were followed to monitor maintenance of remission. As shown, no significant (ns) difference between the two groups was found with respect to maintenance of clinical remission at the end of the follow-up time (600 days).

Figure 3 shows the Kaplan-Meier survival graphs of remission maintenance during a 600 day follow-up observation at the end of active treatment. As shown, the rate of relapse was not significantly different between the GMA and the PSL groups.

### The cumulative dose of corticosteroid in the GMA and the PSL groups

From patients’ case records, we could calculate the total amount of corticosteroid patients had received for the duration of UC in either group. Corticosteroid was PSL in all patients at our hospital. The cumulative amount of PSL before the start of the present investigation was 2556.8mg in the GMA group and 3030.4mg in the PSL group, not significantly different (Table 2). The cumulative amount of PSL during the present study was 0mg in the GMA group and 1122.4mg in the PSL group (P<0.001). All patients in the GMA group were steroid naïve for that flare up.

**Table 2:** Outcomes of the cost analyses for two groups of patients receiving adsorptive granulocyte and monocyte apheresis (GMA) or prednisolone (PSL) at the end of the study (average values are presented)

**Factored item**	**GMA**	**PSL**	**P value**
Days of hospitalization	41.1	41.8	ns
	(n=19)	(n=17)	
Hospitalization cost (euro)*	12739.4	8633.6	P<0.05
	(n=19)	(n=17)	
Cumulative dose of PSL before the treatment (mg)	2457.9	3030.4	**ns**
	(n=24)	(n=17)	
Concomitant dose of PSL during this study (mg)	0 (naïve)	1122.4	P<0.001
	(n=24)	(n=17)	
Cumulative dose of PSL up to the end of study (mg)	2457.9	4152.8	P=0.28
	(n=24)	(n=17)	
Adverse event (%)			
Transient	12.5	11.8	ns
Non-transient	0	35.3	P<0.001

* Converted at 150 yen to the euro. Hospitalization cost includes treatment cost, medication, tests.

### Adverse events and treatment cost

**Figure 4:**
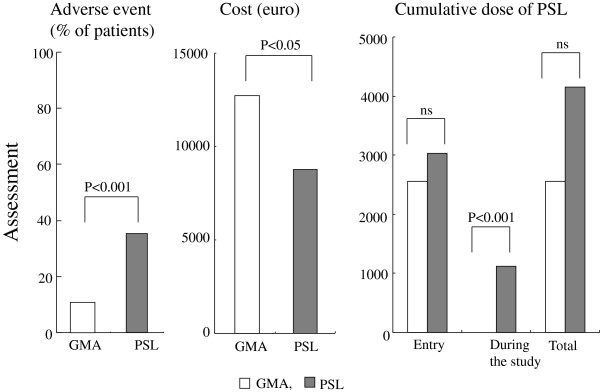
**Assessment of adverse events, medical costs (1€ = 150JPY) and the cumulative dose of prednisolone (PSL) in two groups of patients with active ulcerative colitis (UC) who received GMA or PSL as a treatment intervention.** This figure shows GMA has favourable safety over PSL, while the treatment cost is in favour of PSL.

Figure 4 shows the outcomes of the adverse event assessment, and medical costs (1€ = 150JPY) in the two groups of patients with UC who received GMA or PSL as a treatment intervention. Five adverse events in 3 of 24 patients (12.5%) were reported in the GMA group. These were transient fever, dizziness and light headedness developed towards the end of a GMA session. These events remitted within 3 h without any medication. In the PSL group, 6 of 17 patients (35.3%) developed corticosteroid related adverse events, total 10 events including steroid acne in 2 patients, mild liver disorder in 2 patients, moon face in 2 patients; osteoporosis in 1 patient and Candida oesophagitis in 1 patient Additionally two patients (11.8%) in the PSL group developed nausea just after the administration of PSL. The latter two events resolved within a few hours similar to the events in the GMA group. Regarding the treatment cost in the present investigation, our calculation showed an average cost of 12739.4€ per patient for GMA and 8751.3€ per patient for PSL (P<0.05).

## Discussion

The major focus of this study was on the safety, efficacy, and the medication cost analyses in patients with active UC being treated by GMA as a non-pharmacologic intervention (GMA group) or with the corticosteroid, PSL (PSL group). All included patients had already achieved remission following these interventions and were reviewed retrospectively. This was possible for the fact that all patients were registered at our hospital and had been treated by the physicians who are the authors of this article. Further, for the analyses we undertook in this study, patients who had achieved remission were included to allow us to see the sustainability of remission achieved with these two interventions. The outcomes might be summarised as follows. As the first treatment end point, we considered UC symptoms, which patients consider as the most serious cause of impaired activities and quality of life. These included diarrhoea, rectal bleeding, and abdominal discomfort. The average time to the cessation of at least one of these symptoms was not significantly different between the patients who received PSL and those who received GMA. The second treatment end point we considered was clinical remission**.** Similar to the first end point, the average time to remission was not significantly different between the two groups. Further, the efficacy outcomes in terms of CAI reflected the outcomes we monitored as time to the cessation of major UC symptoms and UC remission. The sustainability of remission was another significant consideration in this study. To see this, we applied the Kaplan-Meier survival analysis of remission maintenance during a 600 day follow-up. The rate of relapse was not significantly different between the two groups.

Regarding the cumulative amount of PSL, in the GMA group, patients had relapsed while not being on a corticosteroid (were steroid naïve for that relapse). These patients received GMA as the only medication for that flare up except that AZA could be continued if a patient had started well in advance of receiving GMA. Nonetheless, we calculated the total amount of PSL patients in the two groups had received for the duration of UC disease. Our calculations did not show any significant difference in the amount of corticosteroid patients had received before the start of the medications factored into this investigation (GMA and PSL). This was because, like the patients in the GMA group, patients in the PSL group had relapsed while not being on a corticosteroid. Further, for this investigation, we ensured that all patients in the GMA group remain corticosteroid free during the follow-up time as well and in the PSL group, the dose of PSL was tapered when patients improve and discontinued when patients achieved remission. Accordingly, the cumulative amount of PSL during the present study was 0mg in the GMA group and 1122.4 mg per patient in the PSL group, clearly showing that GMA spares patients from corticosteroids. Similarly, our safety evaluation showed the data to be very much in favour of GMA with just a few transient and non-serious adverse side effects, which is in line with earlier reports on the safety of GMA in patients with UC [10,12,25-33]. In contrast, over 40% of patients in the PSL group developed adverse events. However our figures from the calculations of the medication cost was in favour of PSL as this is a relatively inexpensive medication as compared with the single use Adacolumns required for GMA. Our impression is that this cost difference is more than offset by the safety of GMA [36].

Following the publication of the first clinical trial of GMA in patients with UC [24], several investigators from Japan, Europe, and the USA [12,27-30,37-43], have reported varying efficacy outcomes ranging from an impressive 85% [12] to a statistically insignificant level [31] Except [31,37,38,41], most other studies did not include a control arm, relying on patients’ disease activity at baseline to serve as a control parameter to judge treatment efficacy [12,27-30]. Among controlled studies, Maiden et al., used GMA to suppress clinical relapse in one arm, while the control arm received no treatment [14]. At the end of a 6-month follow-up, both the relapse rate and time to clinical relapse were significantly better in the GMA arm [14]. Further, intensive GMA involving two sessions per week was found to be more effective than the routinely applied weekly GMA sessions [44]. Likewise, there are reports saying that patients with deep colonic lesions and extensive loss of the mucosal tissue at the lesion sites show very poor response to GMA [12,29,43], while first episode and steroid naïve cases respond well and avoid corticosteroids [12,29,39].

## Conclusions

The results of this study showed that GMA as a non-pharmacologic treatment intervention in patients with UC produced efficacy equivalent to the corticosteroid, PSL and was without safety concern. In view of serious adverse side effects associated with long-term administration of drugs, GMA is very much favoured by patients for its safety profile. In particular, young and elderly patients should benefit most from GMA as a replacement for corticosteroids and other potentially unsafe medications including biologics. However, the results of this study require further support by a prospective controlled study in large cohorts of patients.

## Abbreviations

5-ASA: 5-aminosalicylic acid; CAI: Clinical activity index; GMA: Granulocytes and monocytes apheresis; IBD: Inflammatory bowel disease; PSL: Prednisolone; UC: Ulcerative colitis.

## Competing interest

The authors declare that they have no competing interests.

## Authors’ contribution

1) KT, KK, and HH: Made substantial contribution to the conception, study design and preparation of the manuscript draft; 2) KT, MN, MH: Patient management, generation, collection, assembly, analysis and interpretation of data; 3) KT, MN, MH, KK, and HH: Revision and approval of the final version of the manuscript.

## Pre-publication history

The pre-publication history for this paper can be accessed here:

http://www.biomedcentral.com/1471-230X/13/41/prepub
